# Effect of immunogenetics polymorphism and expression on direct-acting antiviral drug response in chronic hepatitis C

**DOI:** 10.1007/s10238-024-01432-x

**Published:** 2024-08-08

**Authors:** Aya Ismail Abdelaziz, Eman Abdelsameea, Mohamed Abdel-Samiee, Samar E. Ghanem, Sara A. Wahdan, Doaa A. Elsherbiny, Zeinab Zakaria, Samar S. Azab

**Affiliations:** 1 Department of Research and Development, Faculty of Pharmacy, Heliopolis University for Sustainable Development, Cairo, Egypt; 2 Department of Hepatology and Gastroenterology, National Liver Institute, Menoufia University, Shebin El-Kom, Egypt; 3 Department of Clinical Biochemistry and Molecular Diagnostics, National Liver Institute, Menoufia University, Shebin El-Kom, Egypt; 4 Department of Pharmacology and Toxicology, Faculty of Pharmacy, Ain Shams University, Cairo, 11566 Egypt

**Keywords:** Daclatasvir, FOXP3, HCV, IL28B, Sofosbuvir, DAA

## Abstract

**Abstract:**

The prevalence of HCV infection in Egypt has decreased following the introduction of direct-acting antiviral therapy. However, treatment response is influenced by various factors, particularly host immunogenetics such as *IL-28B* and FOXP3 polymorphisms. The current study examined the impact of SNPs in the *FOXP3* gene promoter region on HCV-infected Egyptian patients, along with SNPs in the IL28B gene.This study involved 99 HCV patients who achieved SVR12 after a 12 week DAA treatment while 63 HCV patients experienced treatment failure. IL28B rs12979860 SNP was identified using real-time PCR, while IL28B rs8099917, *FOXP3* rs3761548, and rs2232365 SNPs were analyzed using RFLP-PCR. Serum levels of IL28B and *FOXP3* were quantified using ELISA technique in representative samples from both groups. The IL28B rs12979860 T > C (*P* = 0.013) and *FOXP3* rs2232365 A > G polymorphisms (*P* = 0.008) were found to significantly increase the risk of non-response. Responders had higher *IL28B* serum levels (*P* = 0.046) and lower *FOXP3* levels (*P* < 0.001) compared to non-responders. Regression analysis showed an association between IL28B rs12979860 and *FOXP3* rs2232365 with treatment response, independent of age and gender. A predictive model was developed with 76.2% sensitivity and 91.9% specificity for estimating DAAs response in HCV patients.Our findings confirmed the IL28B rs12979860 T > C and FOXP3 rs2232365 A > G polymorphisms significantly affect DAA treatment response in HCV Egyptian patients. Lower levels of *IL-28B* along with higher levels of FOXP3 are linked to poor response. Our results may lead to new insights into DAA responsiveness contributing to personalized medicine and improving therapeutic decision-making for HCV patients.

**Graphical abstract:**

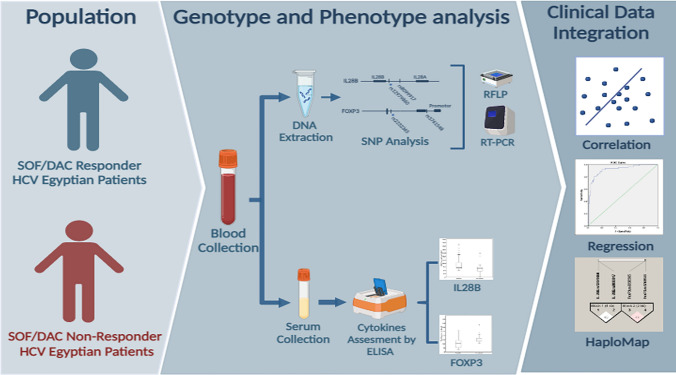

**Supplementary Information:**

The online version contains supplementary material available at 10.1007/s10238-024-01432-x.

## Introduction

Based on the latest estimate of the World Health Organization (WHO), 58 million people have chronic hepatitis C virus (HCV) infection worldwide. Chronic HCV is an urgent worldwide health issue leading to liver cirrhosis which increase the development of hepatocellular carcinoma (HCC), and liver transplantation [1, 2]. Due to the use of poorly sanitized glass syringes for parenteral anti-schistosomal treatment between 1920 and 1980, Egypt was among the Eastern Mediterranean regions with higherrates of HCV infection, with an estimated prevalence of around 14% [3, 4]. Currently, healthcare-related transmission is the main route of HCV infection in Egypt, with approximately 150,000 new infections per year [5]. Among the eight confirmed genotypes of HCV, genotype 4 (GT4) accounts for 91% of HCV-infected Egyptian patients [6–8].

Previously, pegylated interferon (PEG-IFN) and ribavirin (RBV) were considered the gold standard for treating all genotypes of HCV. However, this treatment was non-specific and associated with several adverse events. In contrast to nonspecific treatment with PEG-IFN, the introduction of oral direct-acting antiviral (DAA) therapy has revolutionized the management of HCV patients [2]. DAAs target specific regions of the HCV genome and are categorized as inhibitors of NS3/4A proteases, NS5B nucleoside polymerase (such as Sofosbuvir [SOF]), NS5B non-nucleoside polymerase, and NS5A inhibitors (such as Daclatasvir [DAC]) [9, 10]. Over 90% sustained virological response at 12 weeks (SVR12) has been achieved by DAAs combinations [11–13]. The substantial cost of DAA therapy coupled with the high occurrence of HCV infection in Egypt, leads to animmense economic burden that necessitates adequate cure rates [14]. Moreover, it is critical to address the issue of patients who have failed DAA therapy, making recovery more challenging.

Recent evidence highlights that in addition to the viral factor, host genetic factors may be involved in the success or failure of antiviral therapy and HCV clearance [15]. Three genome-wide association studies (GWAS) have identified single nucleotide polymorphisms (SNPs) near the interleukin 28B (*IL-28B*) gene that are strongly associated with SVR to treatment with pegylated interferon alpha (PEG-IFN-*α*) and/or ribavirinalone. They stated that the most significant polymorphisms of the *IL-28B* gene were rs12979860, rs8099917, and rs12980275 [16–18]. Research on *IL-28B* polymorphisms in various populations has demonstrated that individuals who harbor the CC genotype of the rs12979860 have a better response to DAAs as a treatment compared to other genotypes [19–21].

*IL-28B* is encoded on the interferon lambda (IFN-λ) gene which plays a significant role in the natural antiviral defenses against HCV [22, 23]. The IFN-λs have increased particular attention due to their association with the spontaneous clearance and inhibition of HCV replication via upregulation of the interferon signaling gene (ISG) [24]. Thus far, there have been limited investigations to study the association of *IL-28B* serum levels and the different genotypes of *IL-28B* in chronic HCV patients undergoing treatment with DAAs [25, 26].

Persistent antigen exposure leads to the exhaustion of T cells, which lessens their capacity to control the infection. Additionally, it can lead to regulatory T cell (Treg) activation, which suppresses the immune response. These dysregulations in the immune system contribute to the sustained presence of HCV infection leading to chronicity [27]. Treg activation may also affect the progress of liver disease by maintaining fibrogenesis and inflammatory tissue activity [28]. A pivotal marker of Tregs modulation is the transcription factor known asforkhead box P3 (*FOXP3*). It is a key factor in the expansion of diverse Treg lines and the maintenance of immunoregulation in different pathological conditions that are both autoimmune and infectious. FOXP3-expressing Tregs possess immunosuppressive properties and exert inhibitory effects on various effector immune cells. Therefore, the polymorphism in *FOXP3* and the level of *FOXP3* are vital in regulating immune responses [29, 30]. Given that the *FOXP3* gene plays a crucial role in regulating the gene expression and activating Treg, its promoter region may include significant SNPs [31]. Among these SNPs, the C > T rs3761549, C > A rs3761548, and A > G  rs2232365 SNPs are functionally defined as immunomodulatory [32]. Few studies have investigated *FOXP3* polymorphisms in conditions other than autoimmune diseases.One study proposed that the immunomodulatory effects on individuals with viral hepatitis are influenced by *FOXP3* SNPs, specifically, rs2232365, rs3761549, and rs3761548 [33]. Another recent study investigated the influence of rs3761547 SNP of *FOXP3 *in the inflammatory responses accompanying viral hepatitis [32].

Accordingly, our objective was to enhance our understanding of immune-related genes that may impact a patient’s response to DAA therapy. Specifically, we aimed to examine the effects of *IL-28B* SNPs (rs12979860, rs8099917) and *FOXP3* SNPs (rs2232365 and rs3761548) on the response to Sofosbuvir and Daclatasvir combination therapy in chronic HCV Egyptian patients. Additionally, we evaluated the serum levels of *IL-28B* and *FOXP3* in relation to treatment response and their associations with the studied SNPs.

## Subjects and methods

### Subject recruitment and study design

A retrospective cohort study was conducted, analyzing data from 162 patients with chronic HCV infection who attended the outpatient unit of the Hepatology and Gastroenterology Department, National LiverInstituteHospital, Menoufia University, Egypt from 2021 to 2022.All cases who participated in the study completed a 12 week treatment course of DAC 60 mg/day and SOF 400 mg/day, orally. The cases were then grouped into responders and non-responders based on their sustained virologic response (SVR) at the end of therapy.

Based on the inclusion criteria, a clinical examination and laboratory testing were performed on all selected patients. These tests included liver enzyme level tests such as alanine aminotransferase (ALT), aspartate aminotransferase (AST), total bilirubin, albumin, creatinine, and a complete blood count (including red blood cells (RBCs), white blood cells (WBCs), platelets, and hemoglobin (HB). Additionally, the AST to Platelet Ratio Index (APRI) Score and Fibrosis-4 (FIB-4) were calculated, and the international normalized ratio (INR), alpha-fetoprotein (AFP), serological tests like surface antigen of HBV (HBsAg) and anti-HCV, HCV RNA quantification, and fibro scan for the diagnosis of liver cirrhosis.

The following individuals were excluded; those diagnosed with decompensated liver disease, Child–Pugh B and C cirrhosis, ascites or history of ascites, hepatic encephalopathy or history of hepatic encephalopathy, patients complicated with hepatocellular carcinoma, serum creatinine > 2.5 mg/dL, pregnancy, and poorly controlled diabetes (HbA1c ≥ 8), INR ≥ 1.7, serum albumin < 2.8 g/dL, total serum bilirubin ≥ 3 mg/dL, platelets < 50 000/mm^3^. Additionally, patients with Hepatitis B virus (HBV),  Human Immunodeficiency virus (HIV) co-infections, other liver disease causes, renal impairment,non-adherence to treatment, and those who declined to be enrolled in the study were excluded.

#### Ethics consideration

The protocol of our study follows the ethical guidelines of the Declaration of Helsinki (2013) and has been approved by the Institutional Review Board of the National Liver Institute (NLI IRB 00003143 ; protocol number 00252/2021) and Faculty of Pharmacy, University of Ain Shams (ACUC-FP-ASU.RHDIRB2020110301REC#37). We adhered to the STROBE (Strengthening the Reporting of Observational Studies in Epidemiology) guideline for this observational study.

### Genetic analysis

Peripheral venous blood samples were divided into one vacutainer for serum preparation and one Na-EDTA vacutainer for DNA extraction. Genomic DNA extraction was conducted using a modified method of a previously described protocol [34].

#### Polymorphism genotyping

##### Genotyping of *IL-28B* C/T (rs12979860)

**Fig. 1:**
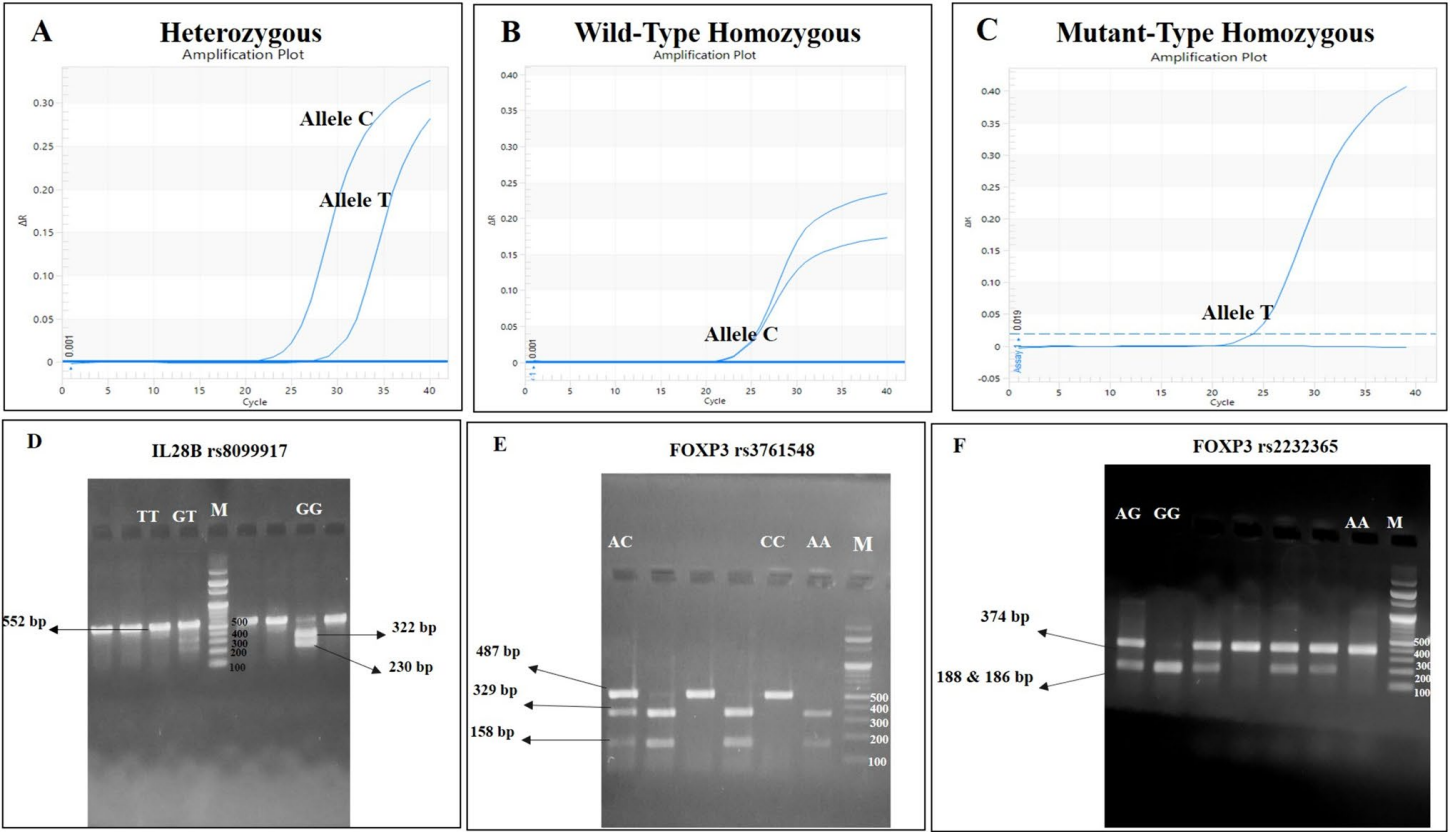
Amplification plots of the genotypes for Interleukin IL28B (rs12979860) polymorphism. The amplification curves are based on the fluorescence intensity variation (ΔRn) according to the number of PCR cycles for the wild-type Heterozygous CT (**A**), Homozygous CC (**B**), and Mutant-Type Homozygous TT (**C**). RFLP of the amplified product of (**D**) For the genotyping IL28B rs8099917, the 552 bp PCR product was digested by BsrDI enzyme. The T allele is not cut by the enzyme where the G allele yields 322 and 230 bp products. (**E**) For the genotyping FOXP3 rs3761548, the 487 bp product was digested with PstI enzyme. The C allele is not cut by the enzyme, whereas the A allele yields 329 and 158 bp products (**F**) For the genotyping FOXP3 rs2232365, the 374 bp PCR product was digested with BsMBI enzyme. The A allele is not cut by the enzyme, whereas the G allele yields 188 and 186 bp products

*IL-28B* genotyping was determined by Eco 48 Real-Time Polymerase chain reaction (PCR) using SYBR green assay (PCRmax Limited, Staffordshire, UK) using 25 ng extracted DNA and with 0.5 µl of each primer (Supp. Table 1) in 20 µl reaction mixture. We followed the PCR protocol by Zakaria et.al.[35]. (Fig. 1A–C).

**Table 1:** Basic characteristics of all subjects included in the study

Variable	Total (N = 162, 100%)	Responder (N = 99, 61.1%)	Non-responder (N = 63, 38.9%)	*P* value
Age (Mean ± SD)	46 ± 12	43.2 ± 12.9	50.1 ± 8.5	< 0.001^**a**^
*Gender N*** (%)**				
Male	63 (38.9%)	31 (31.3%)	32 (50.8%)	0.01^**b**^
Female	99 (61.1%)	68 (68.7%)	31 (49.2%)	
ALT (IU/L**)** Median (range)	39 (11–179)	39 (11–179)	49 (12–344)	0.002^**c**^
AST (IU/L**)** Median (range)	37.5 (11–163.7)	35(16–135)	42 (11–163.7)	0.155
AFP (IU/L**)** Median (range)	3.2 (1.0–5.8)	3.0 (1.0–5.7)	3.6 (1.1–5.8)	0.001^**c**^
Albumin (g/dL**)** (Mean ± SD)	4.20 ± 0.7	4.20 ± 0.8	4.10 ± 0.6	0.371
Total Bilirubin** (**mg/dL**)** Median (range)	0.6 (0.1–1.65)	0.6 (0.1–0.94)	0.65 (0.12–1.65)	0.216
WBCx10^3/mm^3 Median (range)	6.5 (3–13.3)	6.50 (3.00–13.30)	6.70 (3.00–11.40)	0.4
HB(g/dl**)** (Mean ± SD)	13.14 ± 1.6	13.23 ± 1.6	12.9 ± 1.5	0.323
Creatinine (mg/dl**)** (Mean ± SD)	0.81 ± 0.17	0.8 ± 0.2	0.8 ± 0.2	0.216
INR (Mean ± SD)	1.1 ± 0.2	1.1 ± 0.2	1.1 ± 0.1	0.420
Platelets** × **10^3/mm**^**3 Median (range)	252.5 (51–550)	243 (51–550)	269 (144–460)	0.015^**c**^
APRI** Score** Median (range)	0.39 (0.09–3.04)	0.39 (0.12–3.04)	0.42 (0.09–0.91)	0.874
Fibrosis Score (FIB-4**)** Median (range)	1.14 (0.21–10.32)	1.03 (0.26–10.32)	1.16 (0.21–5.06)	0.422
HCV RNA Quantitation IU/ml (Pretreatment**)** Median (range)	1,460,000 (2583–66,000,000	410,000 (2583–26,600,000)	4,200,000 (500,000–66000000)	< 0.001^**c**^

^a^*P* value < 0.05 statistically significant using Student t-test

^b^*P* value < 0.05 statistically significant using Chi Square test

^c^*P* value < 0.05 statistically significant using Mann–Whitney test

*ALT* alanine aminotransferase, *AST* aspartate aminotransferase, *AFP* alpha fetoprotein, *WBC* white blood cells, *HB* Hemoglobin, *INR* international normalized ratio, *APRI AST* to platelet ratio index, *HCV* hepatitis C virus

##### Genotyping of *IL-28B* (rs8099917), *FOXP3* [rs3761548], and *FOXP3* (rs2232365)

Genotyping of *IL-28B* (rs8099917), *FOXP3* intron 1 (rs3761548), and (rs2232365)

polymorphisms were conducted using the polymerase chain reaction-restriction fragment-length polymorphism (PCR–RFLP) method. The primer sequences for forward and reverse are provided in Table S1.The target sequences for each gene were amplified using a thermocycler 006 (a&e™, UK) with 50 ng of extracted DNA and 1 μl of each primer in a 20 μl PCR mixture (TOP simple^TM ^DyeMIX-*nTaq* Polymerase,Enzynomics, INC, South Korea). The calculated amount of PCR product was then digested using the relevant enzyme specified in Table S1 which were purchased from NEW ENGLAND BioLabs_Inc_, MA. The digestion conditions were as follows:

For *IL-28B* G/T (rs8099917): Three μl of PCR product (552 bp in length) was digested using BsrDI enzyme at 65 °C for 30 min then separated on a 1.5% agarose gel stained with 5% ethidium bromide and examined under ultraviolet light (UV transilluminator, PHASE, Germany). The BsrDI digestion yielded 552 bp for the undigested allele G and 322, 230 bp for the T allele as shown in Fig. 1D.

For *FOXP3* A/C (rs3761548): PstuI enzyme was used to digest 5 μl of PCR product (487 bp in length) at 37 °C for 40 min and separated on 1.5% agarose gel stained with ethidium bromide. The PstI digestion of the PCR product yielded 487 bp for the undigested allele A, and 329 and 158 bp for  allele C as shown in Fig. 1E.

For *FOXP3* A/G (rs2232365): A 5 μl aliquot of PCR product (374 bp in length) was digested using 200 nl BsMBI enzyme at 55 °C for 30 min and separated on a 1.5% agarose gel. The BsMBI digestion of PCR products yielded 374 bp for the A allele, whereas for allele G, 188,186 bp fragments were observed as presented in Fig. 1F.

To ensure result repeatability, a 10% sample of the subjects from both groups was genotyped twice, with 100% reproducibility.

### Assessment of human serum levels of FOXP3 and *IL-28B*

For quantitative determination of *IL-28B* and *FOXP3* serum levels, the concentrations of circulating *IL-28B* (Cat.No E0692Hu) and *FOXP3* (Cat.No E4773Hu) in serum were determined using commercially available ELISA kits (Bioassay Technology Laboratory, BT LAB, England), according to the manufacturer’s instructions. The minimum level of detection for *IL-28B* and *FOXP3* were 0.25 ng/L and 0.09 ng/ml, respectively. The developed color reaction was measured at OD 450 units on an ELISA reader (ELx 808, BIO-TEKInstruments, US).

### Sample size calculation

Following the study of Zakaria et al. (2019); the percentage of TT homo-mutant genotype of *IL-28B* rs12979860 in HCV-positive cases is 13%. Assuming a population size of 500 HCV-infected individuals, a minimum sample size of 130 individuals is required with a margin of error of 0.05 and a 95% confidence interval. To compensate for the loss in follow-up; the sample size was increased by 15% to 150 cases. The sample size was estimated using the NQuery statistical package, version 7.0, Los Angeles, CA [35].

### Statistical analysis

Once the data was collected and reviewed, it was coded and entered into the Statistical Package for Social Science (IBM SPSS) version 26. Parametric data were presented as mean and standard deviation, while non parametric data were presented as median with range. Qualitative variables were presented as frequency and percentages. Normality was examined with the application of the Kolmogorov–Smirnov test or Shapiro–Wilk test. Pearson’s Chi-square test was used to examine the relation between qualitative variables. A comparison of quantitative variables between the two groups was done using the Student t-test for normally distributed data. Mann–Whitney test or Kruskal–Wallis test were used for not normally distributed data. Correlation between numerical variables was tested using Spearman-rho correlation. Simple and multiple binary logistic regression was performed with the forward approach to adjust for significant covariates. The receiver operating characteristic curve (ROC) was used to examine the prediction ability of the model and presented as an area under the curve (AUC) with its 95% confidence interval (CI). The odds ratio (OR) with its 95% CI was used for risk assessment. All tests were two-tailed. A *p*-value < 0.05 was considered significant. All the genotype frequencies of the selected SNPs in ourstudy followed the Hardy–Weinberg equilibrium.

We used Haploview 4.1 software [36] to identify haplotype blocks by analyzing the linkage disequilibrium (LD) between polymorphisms. We employed the linkage disequilibrium coefficient (D’). The proportion of haplotypes in each group was compared usinga Chi-squared test.

## Results

### Clinical traits of enrolled subjects

In the present study, 162 participants infected with HCV-GT4 and treated with SOF/DAC combination were enrolled. Ninety-nine Egyptian participants (61.1%) were responders, and sixty-three participants (38.9%) were non-responders. Table 1 provides the summary statistics for the baseline characteristics of the studypopulation.The age mean of all participants was 46 ± 12 years. Of the response group, the mean age was 43.2 ± 12.9 years which was significantly less than the mean ageforthe non-responder group (50.1 ± 8.5, *P* Value < 0.001). The percentages of males and females in the non-responder group were comparable, while the percentage of females was significantly higher than males in the responder group (*P* Value = 0.01). Among the non-responder group, the levels of ALT, AFP, platelets count, and HCV quantitationwere statisticallysignificantlyhigher than the responder group, *P* values = 0.002, 0.001, 0.015, < 0.001, respectively.

### Association of the *IL-28B* and *FOXP3* polymorphisms with response to DAAs in HCV treated subjects

First, we investigated the association between four SNPs—*IL-28B* rs12979860, *IL-28B* rs8099917, *FOXP3* rs3761548, and *FOXP3* rs2232365—and treatment response in 162 HCV-infected subjects treated with (SOF/DAC) for 12 weeks.

Table 2 and Fig. S1 provide an overview of the frequencies of genotypes and alleles for the SNPs in responders and non-responders. For the responder and non-responder groups, each polymorphism was in Hardy–Weinberg equilibrium.

**Table 2:** Genotypic distribution and allele frequencies for *IL-28B* and FOXP3 gene polymorphisms among responder and non-responder groups

SNP	Genotype Frequency	Responder N (%)	Non-responder N (%)	*X*^*2*^			*OR* (95% C.I)		*P* value
				6.77					0.034*
***IL28B* rs12979860**	**CC**	30 (30.3%)	11 (17.5%)						
				**Dominant Model (CC vs CT + TT)**					
	**CT**	53 (53.5%)	32 (50.8%)	3.36			2.06 (0.943–4.479)		0.07
				**Recessive Model (TT vs CC + CT)**					
	**TT**	16 (16.2%)	20 (31.7%)	5.41			0.41 (0.195–0.881)		0.02*
	**C allele (Reference)**	113 (57.07%)	54 (42.86%)	6.228			1.77 (1.129–2.784)		0.013*
	**T allele**	85 (42.93%)	72 (57.14%)						
***IL28B* rs8099917**				5.7					0.062
	**TT**	69 (69.7%)	46 (73.0%)						
				**Dominant Model (TT vs GT + GG)**					
	**GT**	30 (30.3%)	14 (22.2%)	0.21			0.85 (0.421–1.716)		0.65
				**Recessive Model (GG vs TT + GT)**					
	**GG**	0 (0.00%)	3 (4.8%)	NA			NA		NA
	**T allele (Reference)**	168 (84.8%)	106 (84.1%)	0.031			1.06 (0.571–1.956)		0.861
	**G allele**	30 (15.1%)	20 (15.9%)						
**FOXP3 rs3761548**				1.579					0.454
	**CC**	32 (32.3%)	18 (28.6%)						
				**Dominant Model (CC vs AC + AA)**					
	**AC**	39 (39.4%)	29 (46.0%)	0.18		1.19 (0.599–2.381)		0.68	
				**Recessive Model (AA vs CC + AC)**					
	**AA**	28 (28.3%)	16 (25.4%)	0.74			1.38 (0.660–2.886)		0.391
	**C allele (Reference)**	103 (52.02%)	67 (51.59%)	0.006			0.98 (0.63–1.54)		0.939
	**A allele**	95 (47.98%)	59 (48.41%)						
**FOXP3 rs2232365**				6.2					0.045*
	**AA**	41 (41.4%)	38 (60.3%)						
				**Dominant Model (AA vs AG + GG)**					
	**AG**	41 (41.4%)	20 (31.7%)	5.51			0.47 (0.244–0.886)		0.019*
				**Recessive Model (GG vs AA + AG)**					
	**GG**	17 (17.2%)	5 (7.9%)	2.42			2.40 (0.830–6.888)		0.12
	**A allele (Reference)**	123 (62.1%)	96 (76.2%)	6.958			0.51 (0.311–0.845)		0.008**
	**G allele**	75 (37.9%)	30 (23.8%)						

*P* value > 0.05: non-significant

*P value < 0.05: significant

**P value < 0.01: highly significant using Chi-square Test

*X*^*2*^: Chi square value, *OR* odds ratio, *C.I* 95% confidence interval, IL28B: interleukin 28B, FOXP3: forkhead box P3, *NA* not applicable*, SNP* singel nucleotide polymorphism, *N* number

For *IL-28B* rs12979860 polymorphism, the CT frequency exhibited the highest frequency genotype in the study subjects; 53.5% in the responder group and 50.8% in non-responder patients. A comparable pattern was observed in the subjects of CC genotype where responders showed 2.06 times more sensitivity to SOF/DAC treatment compared to non-responders. In contrast, the TT genotype was significantly more resistant to treatment than other genotypes (*P* = 0.02). It was found that the risk allele “T” was the most common (57.14%) among the non-responder group and significantly higher as compared to the responder group (42.93%) with an OR of 1.77 (*p* = 0.013).

Meanwhile, for *IL-28B* rs8099917, the “T” allele frequency was the predominant allele between the studied subjects, with a frequency of 84.8% in responders and 84.1% in non-responders. Yet, the genotypes and allele distributions of this polymorphic variant did not differ significantly across the groups studied.

For the *FOXP3* rs3761548 polymorphism, the “C” allele had the highest percentageamong the two groups, accounting for 52.02% of responders and 53.17% of non-responders. The genotype and allele frequencies of the variations in this polymorphism did not show statistically significant differences.

Furthermore, for *FOXP3* rs2232365 polymorphism, a significant difference was identified in the different genotypes (*P* = 0.045). Table 2 presented that the “A” allele distribution frequencies were 62.12% and 76.19% among the responder and non-responder subjects, respectively. The distribution of *FOXP3* rs2232365 genotypes in responders was as follows: AA and AG were the most predominant genotypes accounting for 41.41% of the population, while the GG genotype represented 17.17%. Contrarily, the frequencies of the AA, AG, and GG genotypes in the non-responder group were 60.32%, 31.75%, and 7.94%, respectively. Interestingly, responders exhibited a significantly higher prevalence of the risk allele "G", with an OR of 0.51 (*P* = 0.008) as compared to non-responders.

**Fig. 2:**
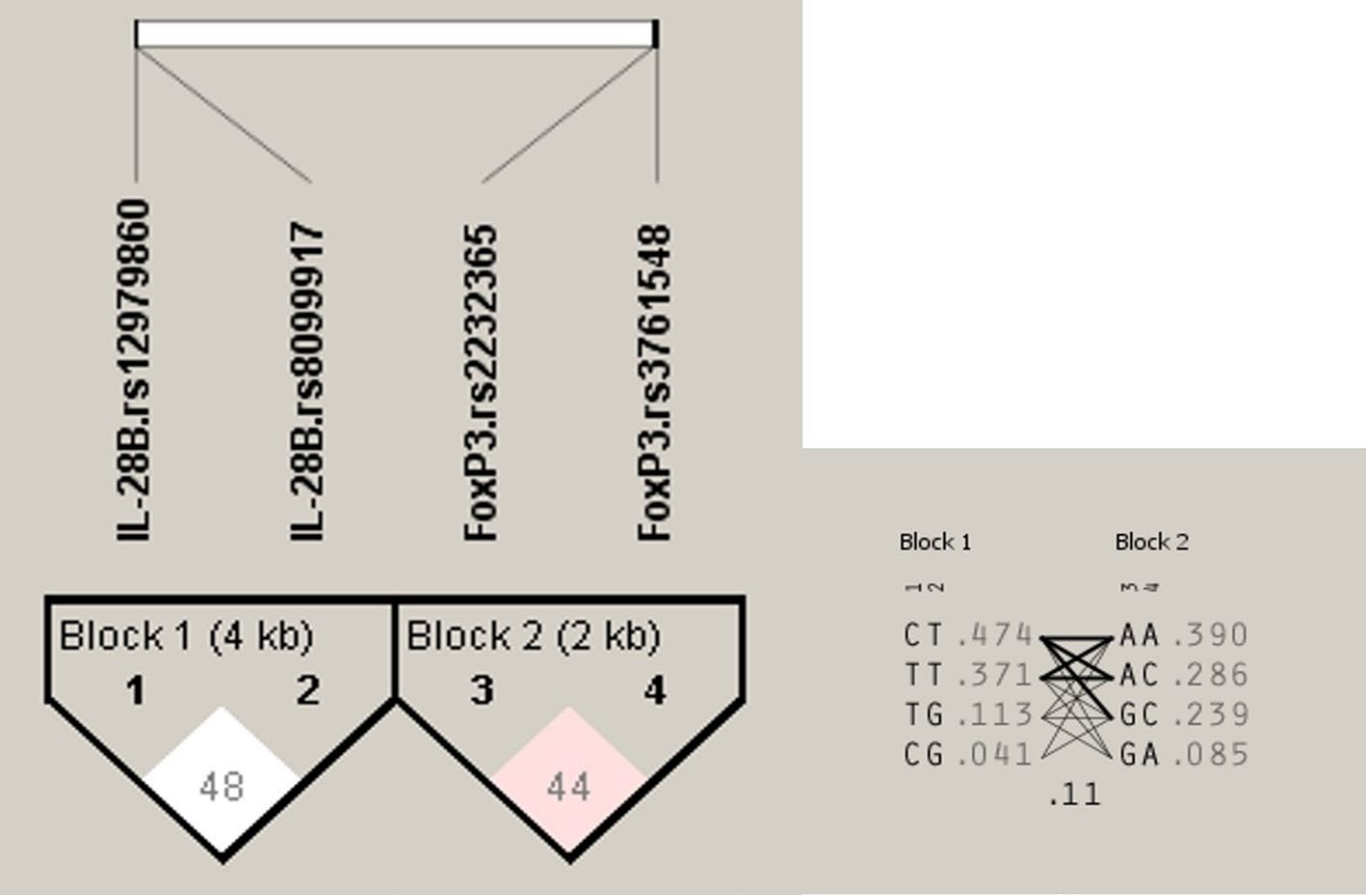
Linkage disequilibrium between genotyped SNPs. The number in each square represents the pairwise LD relationship (r2) between the IL28B (rs12979860, rs8099917) and FOXP3 (rs2232365, rs3761548) SNPs in Egyptian HCV patients treated with Daclatasvir (DAC) 60 mg/day and Sofosbuvir (SOF) 400 mg/day and varying red color represent the linkage disequilibrium values for that pair as measured by D′ (bright red shows high D′). The numbers in the gray-shaded area represent inferred listed in Table 3. The inferred in each haplotype block and the linkage between blocks are shown. The lines between haplotype blocks indicate the linkage frequency with a thick line for > 10% and a thin line for a frequency from 1–10%. Haplotype frequency within each haplotype block is shown next to each haplotype

A haplotype block was allocated to the studied polymorphisms (Fig. 2). It was shown that among HCV patients, the CTAA haplotype predominated. The polymorphic variation in *FOXP3* and *IL-28B* did not exhibit linkage disequilibrium. However, a weak linkage disequilibrium was observed between the two SNPs of *IL-28B* and *FOXP3* (D′: 0.484, r^2^: 0.045/ D′:0.447, r^2^: 0.087; respectively). Table 3 represented the haplotype frequency for each block.

**Table 3:** Haplotypes frequencies for the studied polymorphisms

Haplotype	Frequency (%)	Non-Responders Frequency (%)	Responders Frequency (%)	Chi Square	*P* value
Block 1 (*IL-28B*.rs12979860—*IL-28B*.rs8099917)					
CT	47.4	39.4	52.5	5.309	0.021^*^
TT	37.1	44.7	32.3	5.064	0.024^*^
TG	11.3	12.4	10.6	0.255	0.614
CG	4.1	3.4	4.5	0.237	0.626
Block 2 (FOXP3.rs2232365—FOXP3.rs3761548)					
AA	39.0	39.8	38.5	0.052	0.819
AC	28.6	36.4	23.6	6.181	0.013^*^
GC	23.9	16.8	28.4	5.743	0.017^*^
GA	8.5	7	9.5	0.581	0.446

*P* value > 0.05: non-significant

*P value < 0.05: significant using Chi-square Test

IL28B: interleukin 28B, FOXP3: forkhead box P3

### Relationship between the genotypes of *IL-28B* and *FOXP3* SNPs and their serum levels in response to DAA therapy

**Fig. 3:**
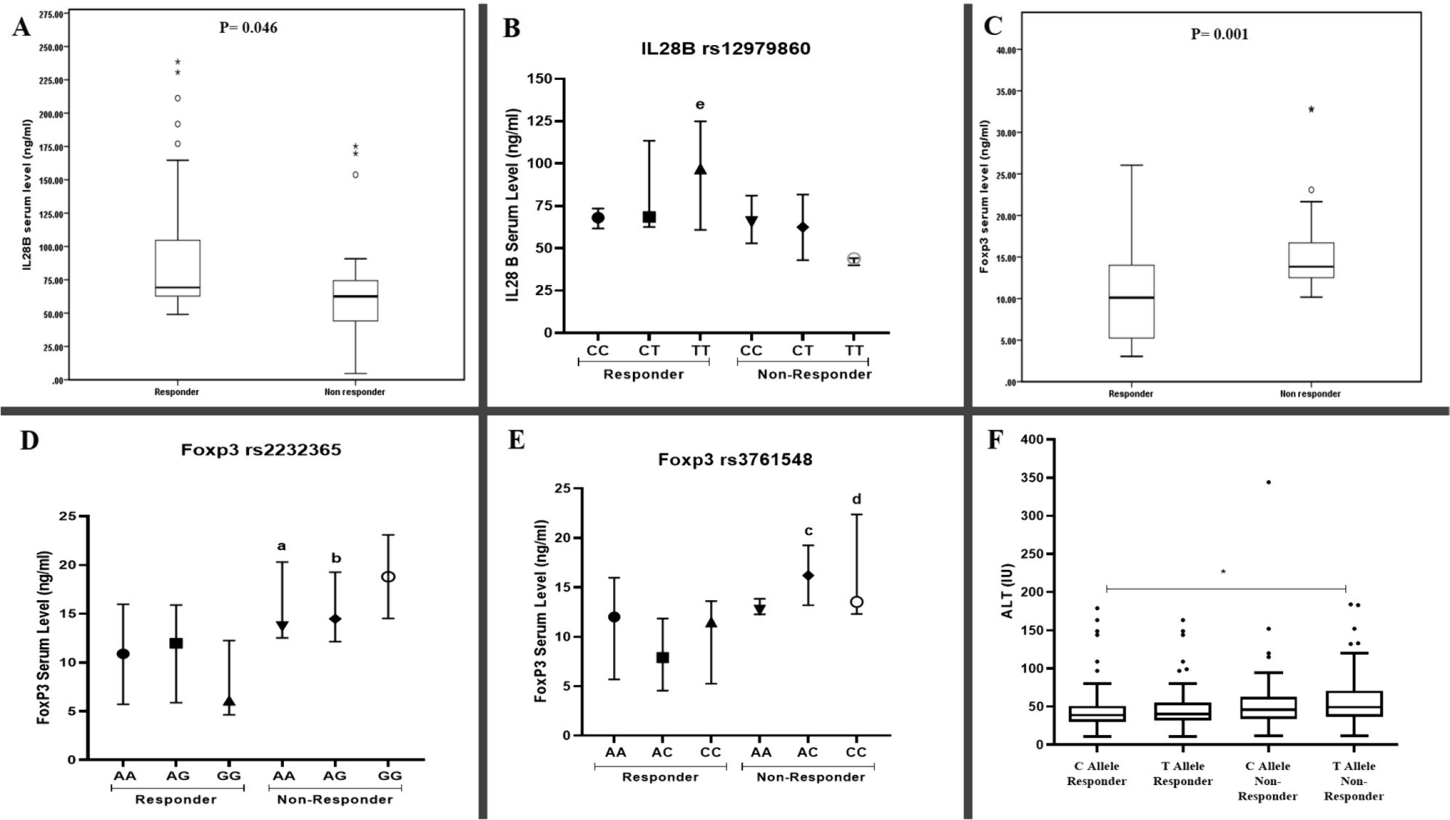
Comparison of **(A)** the serum level of IL28B between responder and non-responder subject; **(B)** IL28B (rs12979860) genotypes against average serum IL28B concentration; **(C)** the serum level of FOXP3 between responder and non-responder subjects and FOXP3 rs2232365 **(D)** and rs3761548 **(E)** genotypes against average serum FOXP3 concentration **(F)** Boxplot for the serum level of ALT among C and T Alleles of responders and non-responders a, b: Statistically Significant of AA and AG genotypes of FOXP3 rs2232365 in non-responder compared to GG genotype in responders using Kruskal-Wallis test followed by Dunn’s test for multiple comparison. c, d: Statistically Significant of AA and AC genotypes of FOXP3 rs3761548 in non-responder compared to AC genotype in responders using Kruskal-Wallis test followed by Dunn’s test for multiple comparison. e: Statistically Significant of TT genotype of IL28B rs12979860 in non-responder compared to TT genotype in responders using Kruskal-Wallis test followed by Dunn’s test for multiple comparison *Statistically Significant between C allele in responder and T allele in non-responder using Kruskal-Wallis test followed by Dunn’s test for multiple comparison

Next, to assess the relationship between serum *IL-28B* and *FOXP3* levels and the various genotypes identified in the studied SNPs, serum samples from both groups were randomly selected for analysis. The results (Table 4), as displayed in Fig. 3A, indicate that there was a statistically significant difference in serum levels of *IL-28B* between patients who responded to DAA therapy and those who did not (*P* = 0.046). Remarkably, a 146.6% rise was detected in serum levels for the TT genotype of *IL-28B* rs12979860 among responders as compared to non-responders (Fig. 3B).

**Table 4:** Interleukin 28B and FOXP3 serum levels among responder and non-responder patients

	Responder	Non-responder	Test value	*P* value
*IL28B* (ng/ml)	69.2 (49.0–238.5)	62.6 (4.7–175.2)	436.0	0.046^*^
FOXP3 (ng/ml)	10.1 (3.0–26.1)	13.8 (10.2–32.9)	352.5	< 0.001^**^

*P* value > 0.05: non-significant

*P value < 0.05: significant

**P value < 0.001: highly significant using Mann–Whitney test

IL28B: interleukin 28B, FOXP3: forkhead box P3

On the other hand, the serum level of *FOXP3* was significantly decreased by 31% in the responder group compared to the non-responder group (*P* < 0.001) as shown in Fig. 3C. Post hoc analysis revealed that responders had the lowest concentration of *FOXP3*, with a median of 6.11 (3.68–12.89), in comparison to non-responders. This was particularly evident in subjects with the *FOXP3* (rs2232365) GG genotype (Fig. 3D). In the same manner, the responder patients with the AC genotype of *FOXP3* rs3761548 showed the lowest level of *FOXP3* with a median of 7.91 (3.6–24.48) compared to other genotypes in non-responder patients (Fig. 3E).

### The association between *IL-28B* and *FOXP3* serum levels and different clinical characteristics in responsive and non-responsive subjects

Higher pretreatment ALT levels have been linked to a slower virological response [37]. Further analysis was conducted to evaluate the correlation between ALT levels and *IL-28B* rs12979860 alleles. Interestingly, the ALT levels were significantly lower in responders with the “C” allele compared to non-responders carrying the “T” allele (Fig. 3F).

Additionally, correlation analysis was conducted to assess the relationship between the serum levels of *FOXP3* and *IL-28B* in terms of several clinical features as shown in Table S2. In the responder group, a noteworthy moderate positive correlation was observed (*P* = 0.001) between the serum levels of *FOXP3* and *IL-28B*, with a correlation coefficient of 0.44. Furthermore, a weak positive correlation was found between the *IL-28B* serum level and HCV quantification (r = 0.35, *P* = 0.007), in addition to platelet count (r = 0.33, *P* = 0.013). No significant associations were found between the serum levels of *IL-28B* or *FOXP3* and other clinical characteristics. Similarly, the results of the correlational analysis in the non-responder group showed that the serum levels of *IL-28B* or *FOXP3* and other clinical characteristics were not associated. Otherwise, there was a moderate negative correlation (r = -0.48, *P* = 0.015) between the serum concentration of *FOXP3* and ALT.

### Univariate and multivariate logistic regression analysis with DAAs treatment response

For variables that showed statistically significant differences between the two groups, a univariate regression analysis was conducted. The purpose of this analysis was to investigate the independent (predictor) and dependent (outcome) variables associated with the response to DAAtherapy. As shown in Table 5, the TT genotype in *IL-28B* rs12979860 was identified to be a risk factor, increasing the possibility of DAA therapy failure by 3.4 times. Furthermore, this mutation remained a reliable predictor of the treatment response regardless of the patient’s gender or age. Conversely, there was nosignificant relationship disclosed between the serum *IL-28B* level and the treatment response.

**Table 5:** Univariate logistic regression model for the prediction treatment response in Egyptian chronic hepatitis C patients

Parameters	Adjusted OR (95% C.I)	*P* Value
*IL28B* rs12979860		
Gender (Male vs. female)	2.42 (1.18–4.95)	0.016
Age	1.06 (1.02–1.09)	0.001
IL28B rs12979860	3.43 (1.49–7.90)	0.004*
FOXP3 rs2232365		
Gender (Male vs. female)	2.17 (1.08–4.37)	0.029
Age	1.05 (1.02–1.09)	0.001
FOXP3 rs2232365	0.327 (0.11–0.99)	0.049*
Serum *IL28B*		
Gender (Male vs. female)	3.68 (1.20–11.29)	0.023
Age	1.03 (0 .99–1.08)	0.137
IL28B	0.99 (0.976–1.005)	0.189
Serum FOXP3		
Gender (Male vs. female)	16.83 (3.78–74.87)	< 0.001
Age	1.01 (0.97–1.06)	0.576
FOXP3	1.29 (1.13–1.47)	< 0.001**
HCV Quantitation		
Gender (Male vs. female)	1.79 (0.80–3.99)	0.157
Age	1.05 (1.01–1.09)	0.008
HCV.DNA Quantitation	1.00 (1.00–1.00)	< 0.001**
ALT		
Gender (Male vs. female)	2.21 (1.09–4.46)	0.027
Age	1.05 (1.01–1.08)	0.005
ALT	1.01 (1.00–1.02)	0.016*
AFP		
Gender (Male vs. female)	2.18 (1.07–4.43)	0.031
Age	1.05 (1.02–1.09)	0.003
AFP	1.63 (1.19–2.21)	0.002*
Platelets		
Gender (Male vs. female)	2.16 (1.07–4.36)	0.032
Age	1.05 (1.02–1.09)	0.001
Platelets	1.01 (1.00–1.01)	0.005*

Bold type indicates statistically significant results

*P* value > 0.05: non-significant

*P value < 0.05: significant

**P value < 0.001: highly significant

*ALT* alanine aminotransferase, *AST* aspartate aminotransferase, *AFP* alpha fetoprotein, *WBC* white blood cells, *HB* hemoglobin, *INR* international normalized ratio, *APRI AST* to platelet ratio index, IL28B: interleukin 28B, FOXP3: forkhead box P3, *HCV* hepatitis C virus

On the contrary, the mutation in *FOXP3* rs2232365 (GG) was found to be associated with the response to DAAs, making it a reliable predictor, with an OR of 0.327 and a statistically significant p-value of 0.049. Interestingly, there is a strong relationship between the serum level of *FOXP3* and the response to DAAs with an OR of 1.29 (95% CI: 1.13–1.47) and a highly significant *P*- value of less than 0.001.

A comparable pattern was observed with HCV quantification, ALT concentration, AFP, and platelet count. These parameters significantly correlated with the responsiveness to DAA therapy, independent of the subject’s age or gender (Table 5).

All the relevant univariate variables described earlier were involved in the multivariate logistic regression analysis, adjusting for confounders, to identify the key predictors of treatment response to DAA therapy. Interestingly as shown in Table 6, the response to DAA therapy was shown to be correlated with a number of variables, including TT variation in *IL-28B* rs12979860, age, gender, ALT, and AFP serum levels, and virological baseline. The following formula was created to develop a prediction equation for estimating the probability (P) of response to DAA therapy in HCV-infected Egyptian patients:

**Table 6:** Multivariate regression analysis for treatment response in Egyptian chronic hepatitis C patients

	Model Coefficient (B)	Adjusted OR (95% C.I)	*P* Value
Gender (Male vs. female)	1.062	2.89 (1.17–7.15)	0.022
Age	0.05	1.05 (1.01–1.09)	0.017
IL28B rs12979860	1.096	2.99 (1.02–8.79)	0.046
HCV Quantitation	0.0000005176	1.00 (1.00–1.00)	< 0.001
ALT	0.014	1.014 (1.00–1.03)	0.031
AFP	0.547	1.728 (1.17–2.55)	0.006
Constant	− 7.408	–	–

*OR* odds ratio, *C.I* 95% confidence interval, *ALT* alanine aminotransferase, *AFP* alpha fetoprotein, IL28B: interleukin 28B, *HCV* hepatitis C virus

***P* = 1/1 + e**^**−**^^(−7.408+0.050*age+0.0000005*HCV DNA+0.014*ALT+0.547*AFP+1.062*gender+1.096*IL−28B^. ^**rs12979860)**^

Where Male = 1, female = 0. *IL-28B* rs12979860 TT = 1, CC + CT = 0.

Receiver operating characteristic curve for the best cut-off point between responder and non-responder groups regarding gender, age, ALT, *IL-28B* rs12979860, AFP, and HCV quantification

**Fig. 4:**
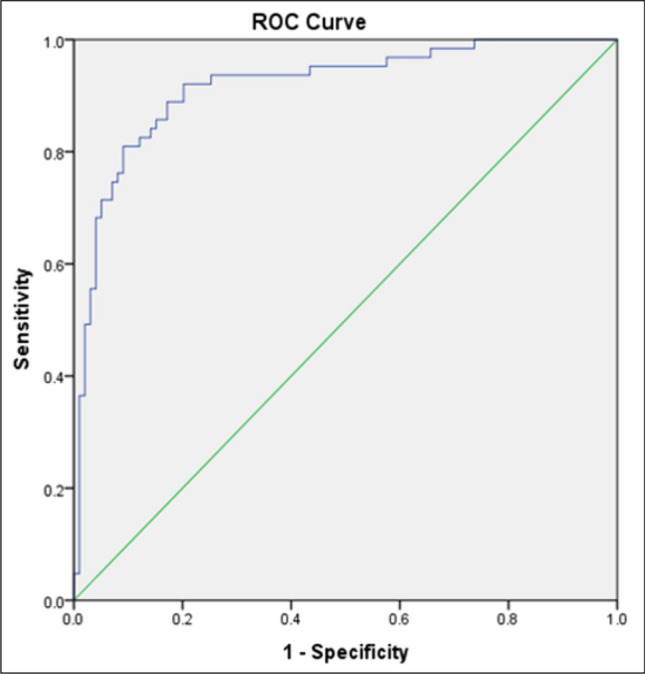
Receiving operating curve (ROC) of the multivariate analysis for DAA treatment response in chronic HCV Egyptian patients treated with Daclatasvir (DAC) 60 mg/day and Sofosbuvir (SOF) 400 mg/day. Area under the curve (AUC) was 0.917 (95% CI = 0.871- 0.963). It is represented a sensitivity of 76.2%, specificity of 91.9%, and total accuracy of 85.8%

The ROC curve was plotted and showed that our predictors have a reliable predictive capacity, with an area under the ROC curve of 0.917 (95% CI = 0.871- 0.963) (Fig. 4). Where the best cut-off value for this model was 5.00, this means that patients above this cut-off value were more likely to exhibit resistance to DAAs treatment. This model presented a sensitivity of 76.2%, specificity of 91.9%, and total accuracy of 85.8%.

## Discussion

The introduction of DAA therapy has changed the epidemiological impact of HCV infection management in the Middle East and North Africa (MENA) region [38]. However, HCV-infected patients exhibit varying responses to DAA therapy, and some may not derive any benefit from it [15]. Consequently, it is imperative to explore the factors that may influence the disease and treatment outcomes. Besides the cost burden of DAAs, predicting disease progression, effectively treating patients, and accurately assessing risks and benefits would pave the way toward personalized medicine for HCV patients. In the present study, it was shown that the response to DAA therapy is reduced in HCV Egyptian patients with IL28B rs12979860 T > C and FOXP3 rs2232365 A > G variants, as well as lower serum IL28B and higher serum FOXP3 levels.

Multiple studies have revealed a significant influence of *IL-28B* polymorphic variations on the treatment outcomes of chronic hepatitis C individuals [17, 39, 40]. Subsequently, several investigators have published similar observations in different populations at different positions of the *IL-28B *gene [26, 35, 41–44]. In particular to the MENA region, the *IL-28B* rs12979860 SNP was found to predict the consequence of HCV genotype 3a infection in the Iranian population, along with the *IL-28B* rs8099917 and *IL-28B* rs8103142 SNPs among HCV GT4-infected Egyptians [26, 35, 45]. A Tunisian study further confirmed the significance of *IL-28B* rs12979860 variation in predicting treatment response and toxicity [46].The same association results were reported among Caucasians, African Americans, and Hispanics [41, 43, 44].

In the era of oral interferon-free therapy,several studies have attempted to determine the impact of *IL-28B* SNPsin DAA treatment response [47]. Therefore, in this study, we explored the treatment outcome of two different SNPs within *IL-28B* among chronic HCV Egyptian patients receiving SOF/DAC combination therapy. In addition to *IL-28B* polymorphisms, we also expanded our investigation to revealvariations in the promoter region of the *FOXP3* gene. It is worth noting that polymorphisms in the *FOXP3* gene can affect the expression of the *FOXP3* protein, leading tochanges in the normal activation of Treg cells [27]. Accordingly, it is crucial to conduct studies to assess the traits of immunogenetic interactionsrelated to disease in various contexts to advance our knowledge and pave the way for personalized medicine.

Recent studies have suggested that the *IL-28B* gene polymorphic variations significantly influence the treatment response of individuals infected with HCV who are receiving DAA therapy. *IL-28B* gene polymorphisms, specifically the SNPs rs12979860 and rs8099917 on chromosome 19, are predictiveof viral eradication after 12 weeks of therapy [19, 48, 49]. In the current study, we revealed that the *IL-28B* rs12979860 “T” allele was approximately twice as likely to be a predisposing factor for non-responders among Egyptian HCV patients. Patients with favorable response genotypes (CC or CT) are more susceptible to respond well to DAAs treatment compared to those with the resistant response genotype (TT). Additionally, we confirmed that the *IL-28B* rs12979860 “T” allele remains a reliable predictor of the responsiveness of DAA therapy, despite the patient’s gender or age. In agreement with our findings, the first case–control study concluded that *IL-28B* rs12979860 is an effective marker for predicting treatment response. The CT genotype had the highest response rate at 62.5%, followed by 30% in CC and 7.5% in TTgenotypes among Egyptian patients with GT4 [50]. However, their findings need to be confirmed, by further research, due to the small sample size and the use of only one DAAs regimen (SOF/RBV) in their study. Our observation is consistent with the results reported by El-Garawani in 2020 which showed a noteworthy rise in the percentage of the CT genotype of *IL-28B* rs12979860 among responders as compared to non-responders receiving SOF/DAC. Additionally, non-responders had a significantly higher frequency of TT genotypes (42.85%) compared to responders [20]. The same results were reported in 131 chronic HCV Egyptian patients with genotype 4 who received SOF and DAC with or without RBV therapy for 3 months [49].

Several researchers have examined the *IL-28B* rs8099917 polymorphism in chronic viral hepatitis, however, this study represents an initial exploration ofthe impact of this SNP within the Egyptian HCV population receiving the SOF 400 mg/day and DAC 60 mg/day regimen [16, 51]. In an Indian cohort study investigating the impactof IFNL3 in genotype 3 chronic HCV patients receiving SOF/DAC combination, they found that TT (major) genotype of *IL-28B* rs8099917 were linked to best SVR rates and GG (minor) genotype of *IL-28B* rs8099917 were more predominant in relapses or non-responses of this regimen [19]. In line with these results, a study conducted on chronic HCV GT4 Egyptian patients who were treated with a combination of PEG-IFN and oral RBV showed that the TT genotype of *IL-28B* rs8099917 was linked to achieving SVR [52]. Conversely, the present observation revealed that in HCV Egyptian patients, the SOF/DAC combination treatment response was not influenced by the mutation in *IL-28B* rs8099917. These results could potentially be attributed to variances in *IL-28B* gene polymorphisms and ethnicity where *IL-28B* polymorphisms accounted for just 50% of inter-individual variability in SVR that were attributed to ethnic differences and only 15% of HCV infection spontaneous clearance [17, 53]. In a recent meta-analysis, the probability of successful SVR was greater for individuals with the CC genotype of rs12979860 CC genotype compared to those with the TT genotype of rs8099917. The positive predictive value of the CC genotype of rs12979860 was 76% whereas that of the TT genotype of rs8099917was 71% [54]. Multiple pieces of evidence emphasized that the existence of the “G” variant allele on the *IL-28B* rs8099917 gene may be regarded as a risk allele for persistent HCV infection rather than predicting treatment response [19, 51]. Alternatively, a cohort study of Australian individuals with chronic HCV genotype 1 who were treated with PEG-IFNα/RBV combination therapy reported an association between SVR and the *IL-28B* rs8099917 genotypes [16]. The current study hypothesized that the contradiction in results could be attributed to the variations in HCV genotype and host ethnicity.

It is interesting to note that the ALT levels, which are known to follow treatment response, were also found to be correlated with the *IL-28B* genotypes. Patients with high inflammatory activity typically have elevated liver enzyme levels such as ALT and AST [55]. The present study found that patients with the favorable “C” allele for *IL-28B* rs12979860 had reduced ALT levels. This reduced level of ALT in our investigation has been accompaniedby a favorable response to DAA therapy. This results in compliance with a recent retrospective case–control analysis reporting that abnormal ALT after SVR following treatment with DAAs is rare [56]. In line with the current results, a recent study revealed that surpassing ALT or AST levels after DAA therapy may indicate treatment failure in mild liver disease [37].

Enduring HCV infection is manifested by weak and impaired cellular immune responses due to dysfunctional dendritic cells. These cells play a vital role in influencing the infection outcome by releasing IFNs and triggering the activation of T-lymphocytes [57–59]. Nevertheless, the mechanism of how polymorphisms in the region of *IL-28B* genes affect treatment responses has remained elusive. The present study hypothesized that as a polymorphism located 3 kb upstream of thepromoter region for the *IL-28B* gene, it may theoretically influence IFN-lambda genes.Therefore, the present study investigated the *IL-28B* serum level in responder and non-responder patients and showed a significant difference between the two groups. It is worth noting that patients who carried the TT genotype of *IL-28B* rs12979860 and still responded to the treatment exhibited elevated levels of *IL-28B* compared to those who remained non-responders.These findings supported the results reported by Langhans et al. (2011) who found a correlation between serum levels of *IL-28B* and the “C” allele of *IL-28B* rs12979860 SNP [60]. Additionally, two Iranian studies have quantified that the expression level of *IL-28B* mRNA increased in HCV-GT1 patients treated with PEG-IFNα /RBV compared to untreated patients. Additionally, the serum level of *IL-28B* in HCV-GT4 patients was higher compared to subjects who achieved immediate clearance [26, 61]. It is well established that IFN λ ultimately exerts its functions through the upregulation of interferon signaling genes, leading to the control and eradication of infection [24]. It is rationally accepted that patients who respond to DAA combination therapy typically have higher concentrations of *IL-28B* in their serum [62]. Accordingly, our findings suggest that low *IL-28B* levels may predispose patients to resist DAAs treatment.

In chronic infections, continuous stimulation by the antigen contributes toexhaustion and dysfunction of T cells, which results in a lessened immune response against the virus [63]. In this scenario, Regulatory T cells (Treg) are the key regulator in sustaining the infection by easing the viral antigens to evade the immune system through the suppression of antiviral responses. *FOXP3*, a transcription factor for Treg cells, is a crucial marker for the development and function of Treg cells [27]. Consequently, the current study aimed to investigate the impact of SNPs in the promoter region of the *FOXP3* gene on *FOXP3* expression levels, which in turn influence Treg cell activation [64, 65]. There is evidence to suggest that the *FOXP3* gene’s ‘‘G’’ allele increases the risk of developing an autoimmune disease. This allele is linked to reduced *FOXP3* expression, which in turn leads to an imbalance in the immune system [66, 67]. In the present study, we investigated two SNPs, rs2232365 and rs3761548, in the promoter of the *FOXP3* gene. We found that the “G” allele in *FOXP3* rs2232365 is considered to have a protective effect in HCV patients who respond to DAAs. On the contrary, the presence of the *FOXP3* rs3761548 polymorphism did not influence the response to DAAs in the studied groups. Strikingly, the individuals who did not respond to DAA therapy showed significantly elevated levels of *FOXP3* in their serum compared to those who responded to the therapy. In addition, the serum levels of *FOXP3* for the rs2232365 AG and AA genotypes along with the rs3761548 AC and CC genotypes elevated in the non-responder individuals. It is implicated that the increased levels of *FOXP3* may initiate a robust hepatic regulatory response potentially leading to a lessening in the immune response against the virus. Consequently, this might ultimately lead to viral reactivation and the continuation of chronic infection [68, 69].

As previously reported, SOF and DAC inhibit HCV replication by targeting RNA polymerase NS5B and NS5A non-structural protein, respectively [70]. Accordingly, *FOXP3* regulates Treg cell function and may suppress antiviral immune responses; moreover, its increased expression in some chronic HCV patients may contribute to a reduced ability to control the viral infection and resistance to DAAs treatment [71]. Limited research has explored the factors that regulate Treg cells in contexts other than autoimmune diseases. One study suggested that *FOXP3* SNPs (rs2232365, rs3761549, and rs3761548) may have an immunomodulatory influence in patients with chronic viral hepatitis. The results indicate that variations in the *FOXP3* gene may play a role in histopathological sequelae observed in the liver following viral infection. Furthermore, elevated levels of liver enzymes and a reduction in viral count were detected in subjects with the GG genotype of rs2232365 *FOXP3* [33]. In a recent study, the contribution of Treg cells in the inflammation induced by viral hepatitis has revealed a noteworthy implication of the *FOXP3* SNP rs3761547 T > C. It has displayed a significant influence on the progression of hepatic fibrosis induced by viral hepatitis [32]. An additional investigation reported that the elevation of *FOXP3* expression is linked to the polymorphic variant A allele in *FOXP3* rs2232365 which potentially leads to an increase in the risk of tuberculosis [72]. This may explain the observed relationship between *FOXP3* serum levels and the DAAs treatment responsiveness, which could be clinically significant for managing HCV patients. Further research is needed to confirm these findings.

The molecular interaction between *IL-28B* and *FOXP3* in HCV in the era of DAAs therapy is still under investigation. Nevertheless, *IL-28B* has been anticipated to decrease the appearance of CD4^+^CD25^+^*FOXP3* ^+^Treg cells during DNA vaccination, thereby modulating adaptive immune responses [73]. Additionally, in murine models, *IL-28B* treatment has been shown to reduce Treg cells and improve antitumor immunity [73]. In the current study, we investigated a weak association between levels of *IL-28B* and *FOXP3* among HCV patients. Further research is required to explore the effect of DAAs on these variables and the relationship between *IL-28B* and *FOXP3* in HCV infection.

The current prediction model observed that being male, increased age, high levels of ALT, higher AFP, a high HCV viral load, and the presence of the T risk allele of *IL-28B* rs12979860 were associated with DAAs resistance in multivariate logistic regression analysis, with 76.2% sensitivity and 91.9% specificity. In other words, the ROC curve analysis demonstrated the utility of the *IL-28B* rs12979860 polymorphism, with a cut-off value of 5, as a predictor of the responsiveness to DAAs therapy in HCV-infected Egyptian patients.

## Conclusion

The present study concludes that rs12979860 C˃T *IL-28B* and rs2232365 G˃A *FOXP3* polymorphisms are regarded as favorable alleles determining the response to SOF/DAC therapy for HCV in Egyptian patients. Furthermore, we have revealed for the first time that the response to DAA combination therapy is correlated with the serum level of *IL-28B* and *FOXP3*. Hence,they can be utilized as a predictive measure to assessthe response to therapy. Until an approved HCV vaccine is developed, it is crucial to continue comprehensive research efforts aimed at reducing the risks associated with HCV treatment resistance. Therefore, our study enhances our understanding of the relationship between genotype and phenotype in the variability of response to DAAs among HCV patients.

It is important to consider that the current study was conducted in an Egyptian HCV-infected population receiving one regimen of DAAs. Hence, future research is needed to assess the generalizability of our findings in different contexts. Also, the present study is subject to limitations primarily due to the investigation of only two SNPs among the genes attributed to Treg functions. Regarding the complexities observed in immune responses, it is recommended that other SNPs of this gene be investigated to clarify the interactions between the pathogen and the host.

## Supplementary Information

Below is the link to the electronic supplementary material.

Supplementary file1 (DOCX 50 KB)

Supplementary file2 (JPG 164 KB)

## Data Availability

The datasets generated during and/or analysed during the current study are available from the corresponding author on reasonable request.
