# Supplementary material for: Effect of immunogenetics polymorphism and expression on direct-acting antiviral drug response in chronic hepatitis C
Source: Clin Exp Med. 2024 Aug 8;24(1):184. doi: 10.1007/s10238-024-01432-x (PMC11310263; doi:10.1007/s10238-024-01432-x)
Supplement: Supplementary file 1 — Supplementary file1 (DOCX 50 KB) [file 10238_2024_1432_MOESM1_ESM.docx]

| **Supp. Table (1): Primers Sequences for the studied Single Nucleotide Polymorphism** | | | | | | | | | | | | |  |
| --- | --- | --- | --- | --- | --- | --- | --- | --- | --- | --- | --- | --- | --- |
| **SNP** | **Primer name** | **Sequence** | | | **Allele** | | **T_m_** | | **Restriction enzyme** | | **Fragment size** | **Reference** | |
| **IL28B rs12979860** | Rs-860-F | GCTTATCGCATACGGCTAGGC | | | C/T | | 60°C | | ------- | | ------- | (35) |  |
|  | Rs-860-R  (**C** Allele) | GCAATTCAACCCTGGTTC**G** |  |  | |  | |  | |  |  |  |  |
|  | Rs-860-R  (**T** Allele) | GCAATTCAACCCTGGTTC**A** | | |  | |  | |  | |  |  |  |
| **IL28B**  **rs8099917** | Rs-917-F | CCCACTTCTGGAACAAATCGTCCC | | | G/T | | 59°C | | BsrDI | | 552  322, 230 | (72) |  |
|  | Rs-917-R | TCTCCTCCCCAAGTCAGGCAACC | | |  |  |  |  |  |  |  |  |  |
| **FOXP3**  **rs3761548** | Rs-548-F | GCCCTTGTCTACTCCACGCCTCT | | | A/C | | 63ºC | | PstI | | 487  329,158 | (73) |  |
|  | Rs-548-R | CAGCCTTCGCCA ATACAGAGCC | | |  |  |  |  |  |  |  |  |  |
| **FOXP3**  **rs2232365** | Rs-365-F | TGGAGGGCTTTCAAGGTGAGGA | | | A/G | | 59⁰C | | BsMBI | | 374  188,186 | (74) |  |
|  | Rs-365-R | GGGGAGTTGGATTGGGTGCA | | |  |  |  |  |  |  |  |  |  |
| T_m_:Melting Temperature, SNP: Single Nucleotide Polymorphism, IL28B: Interleukin 28B, FOXP3: forkhead box P3, Rs: Reference Sequence, F: Forward Primer, R: Reverse Primer | | | | | | | | | | | | |  |

| **Supp. Table (2) Correlation Analysis Correlation Analysis of IL28B and FOXP3 Serum Levels with different factors** | | | | |
| --- | --- | --- | --- | --- |
| **(A): Responder Subjects** | | | | |
|  | **IL28B Serum Level** | | **FOXP3 Serum Level** | |
|  | **r** | **P Value** | **r** | **P Value** |
| **IL28B Serum Level** | - | - | - | - |
| **FOXP3 Serum Level** | **0.436** | ***0.001^**^*** | - | - |
| **HCV DNA Quantification** | **0.355** | ***0.007^**^*** | 0.090 | 0.496 |
| **ALT (IU/L)** | 0.047 | 0.731 | 0.197 | 0.731 |
| **AST (IU/L)** | 0.045 | 0.743 | 0.2 | 0.129 |
| **AFP (IU/L)** | 0.067 | 0.621 | 0.067 | 0.612 |
| **Albumin (g/dL)** | 0.032 | 0.813 | 0.006 | 0.962 |
| **Total bilirubin (mg/dL)** | -0.022 | 0.874 | -0.167 | 0.205 |
| **WBCx10^3/mm^3** | 0.003 | 0.982 | -0.086 | 0.518 |
| **HB(g/dl)** | 0.184 | 0.174 | 0.050 | 0.710 |
| **Creatinine (mg/dl)** | -0.013 | 0.927 | -0.143 | 0.279 |
| **INR** | 0.008 | 0.954 | 0.096 | 0.467 |
| **Platelets x10^3/mm^3** | **0.330** | ***0.013^*^*** | 0.099 | 0.456 |
| **APRI Score** | -0.159 | 0.243 | 0.053 | 0.689 |
| **Fibrosis Score** | -0.069 | 0.613 | 0.009 | 0.947 |

| **(B): Non-Responder Subjects** | | | | |
| --- | --- | --- | --- | --- |
|  | **IL28B Serum Level** | | **FOXP3 Serum Level** | |
|  | **r** | **P Value** | **r** | **P Value** |
| **IL28B Serum Level** | - | - | - | - |
| **FOXP3 Serum Level** | 0.226 | 0.312 | - | - |
| **HCV DNA Quantification** | -0.094 | 0.678 | -0.248 | 0.232 |
| **ALT (IU/L)** | -0.189 | 0.399 | **-0.480** | ***0.015^*^*** |
| **AST (IU/L)** | -0.369 | 0.091 | -0.328 | 0.110 |
| **AFP (IU/L)** | -0.232 | 0.298 | -0.169 | 0.418 |
| **Albumin (g/dL)** | 0.212 | 0.343 | 0.142 | 0.498 |
| **Total bilirubin (mg/dL)** | 0.024 | 0.915 | -0.228 | 0.274 |
| **WBCx10^3/mm^3** | 0.340 | 0.122 | 0.282 | 0.172 |
| **HB (g/dl)** | 0.251 | 0.259 | -0.282 | 0.171 |
| **Creatinine (mg/dl)** | -0.129 | 0.567 | -0.067 | 0.752 |
| **INR** | -0.252 | 0.257 | 0.012 | 0.956 |
| **Platelets x10^3/mm^3** | -0.041 | 0.855 | -0.025 | 0.904 |
| **APRI Score** | -0.383 | 0.078 | -0.322 | 0.117 |
| **Fibrosis Score** | -0.223 | 0.320 | -0.195 | 0.349 |

P value > 0.05: non-significant; *P value < 0.05: significant; **P value < 0.01: highly significant. Bold type indicates statistically significant results.

ALT: alanine aminotransferase, AST: aspartate aminotransferase, AFP: Alpha fetoprotein, WBC: white blood cells, HB: Hemoglobin, INR: international normalised ratio, APRI: AST to Platelet Ratio Index, IL28B:interleukin 28B, FOXP3: forkhead box P3, HCV: Hepatitis C Virus

**M**

**CC AA**

**AC**
