# Supplementary figures and images for: Effect of immunogenetics polymorphism and expression on direct-acting antiviral drug response in chronic hepatitis C
Source: Clin Exp Med. 2024 Aug 8;24(1):184. doi: 10.1007/s10238-024-01432-x (PMC11310263; doi:10.1007/s10238-024-01432-x)

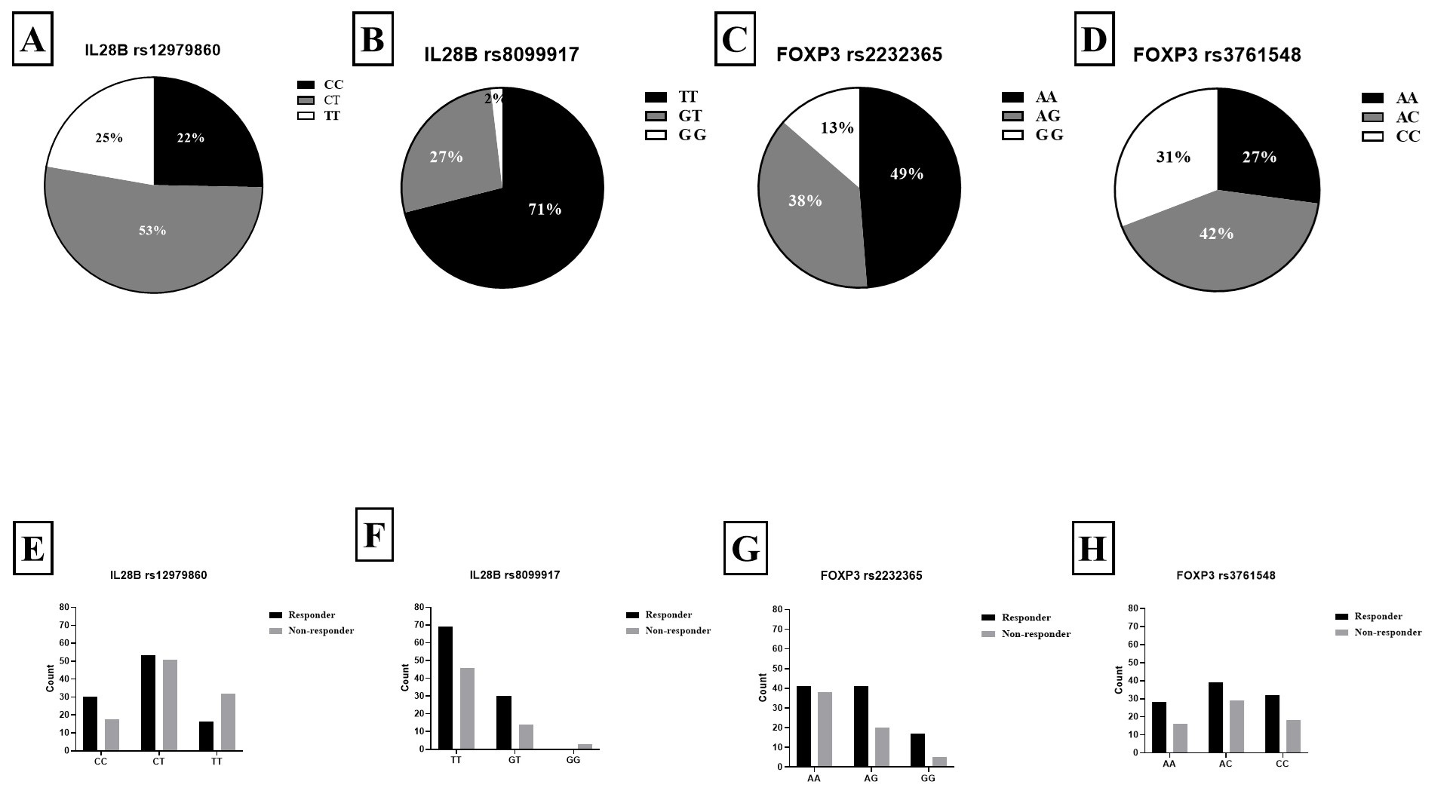

Supplement: Supplementary file 2 — Supplementary file2 (JPG 164 KB) [file 10238_2024_1432_MOESM2_ESM.jpg]
